# Recurrent presacral tailgut cyst with mucinous adenocarcinoma and metastasis

**DOI:** 10.1016/j.radcr.2025.04.128

**Published:** 2025-05-26

**Authors:** Iyad Al Jada, Majd Oweidat, Mohammad Khaleel, Omar Harb, Ursula Abu Nahla, Rahaf Bleibel, Mai Arafeh, Ammar W.M. Hassouneh

**Affiliations:** a College of Medicine, Hebron University, Hebron, West Bank, Palestine; b Department of Surgery, Princess Alia Hebron Governmental Hospital, Hebron, West Bank, Palestine; c Department of Radiology, Princess Alia Hebron Governmental Hospital, Hebron, West Bank, Palestine; d Department of Radiology, Al-Mezan Specialty Hospital, Hebron, West Bank, Palestine

**Keywords:** Tailgut cyst, Metastasis, Mucinous adenocarcinoma, Malignant degeneration

## Abstract

Tailgut cysts (TGCs) are rare congenital lesions arising from incomplete regression of the embryonic hindgut, typically located in the presacral space and more common in females. Although often benign, these cysts carry a risk of malignant transformation. We report a case of a woman in her early 50s who presented with abdominal pain, urinary retention, and tenesmus. Initial imaging revealed a large presacral cystic lesion, and incomplete surgical excision confirmed benign histology. However, the patient returned 6 months later with recurrent symptoms and new-onset lower limb weakness. Repeat imaging showed a recurrent cystic lesion with liver and bony metastases. MRI showed peripheral nodular enhancement of the lesion and enhancing bone lesions. A liver biopsy confirmed mucinous adenocarcinoma. Despite chemotherapy, the disease progressed rapidly, and the patient passed away within 2 months. This case highlights the potential for malignant degeneration in TGCs, which, though rare, carries significant morbidity. Complete surgical resection and follow-up are critical to prevent recurrence and malignancy risk. Further research is needed to study the malignant transformation predictors of this rare entity.

## Introduction

Presacral tailgut cysts (TGC), also known as retrorectal cystic hamartomas, are uncommon congenital anomalies resulting from incomplete regression of the embryonic hindgut. Under normal circumstances, the tailgut undergoes involution by the sixth week of gestation. However, when this process fails, a tailgut cyst forms, and it is typically located in the presacral space [1,2]. TGCs primarily affect females, with a reported female-to-male ratio ranging from 3:1 to 9:1 [3].

TGCs may range from asymptomatic incidental findings to prolapsing cysts, which can be misdiagnosed as hemorrhoids. Symptoms, when present, include constipation, incomplete rectal evacuation, dysuria, and urological or neurological issues [4]. TGCs are primarily diagnosed through imaging and histopathological examination [4]. Surgical intervention is the cornerstone of treatment, primarily to address complications like malignant transformation, infection, or fistula formation [5].

We present the case of a woman diagnosed with a recurrent TGC who underwent incomplete excision and subsequently developed metastatic mucinous adenocarcinoma.

## Case presentation

A female patient in her early 50s presented to our tertiary care unit with a 2-month history of progressive lower abdominal pain, urinary retention, and tenesmus. She had no significant past medical or surgical history, and her family history revealed no notable malignancies. She reported no previous episodes of similar symptoms. On physical examination, posterior fullness with palpable cystic edges was noted during a digital rectal examination (DRE), accompanied by tenderness in the lower abdominal region. The remainder of the physical examination was unremarkable.

Initial laboratory tests showed normal levels of carbohydrate antigens (CA), including CA 19-9, CA-15-3, and CA-125. However, her carcinoembryonic antigen (CEA) level was mildly elevated at 5 ng/mL.

**Fig. 1:**
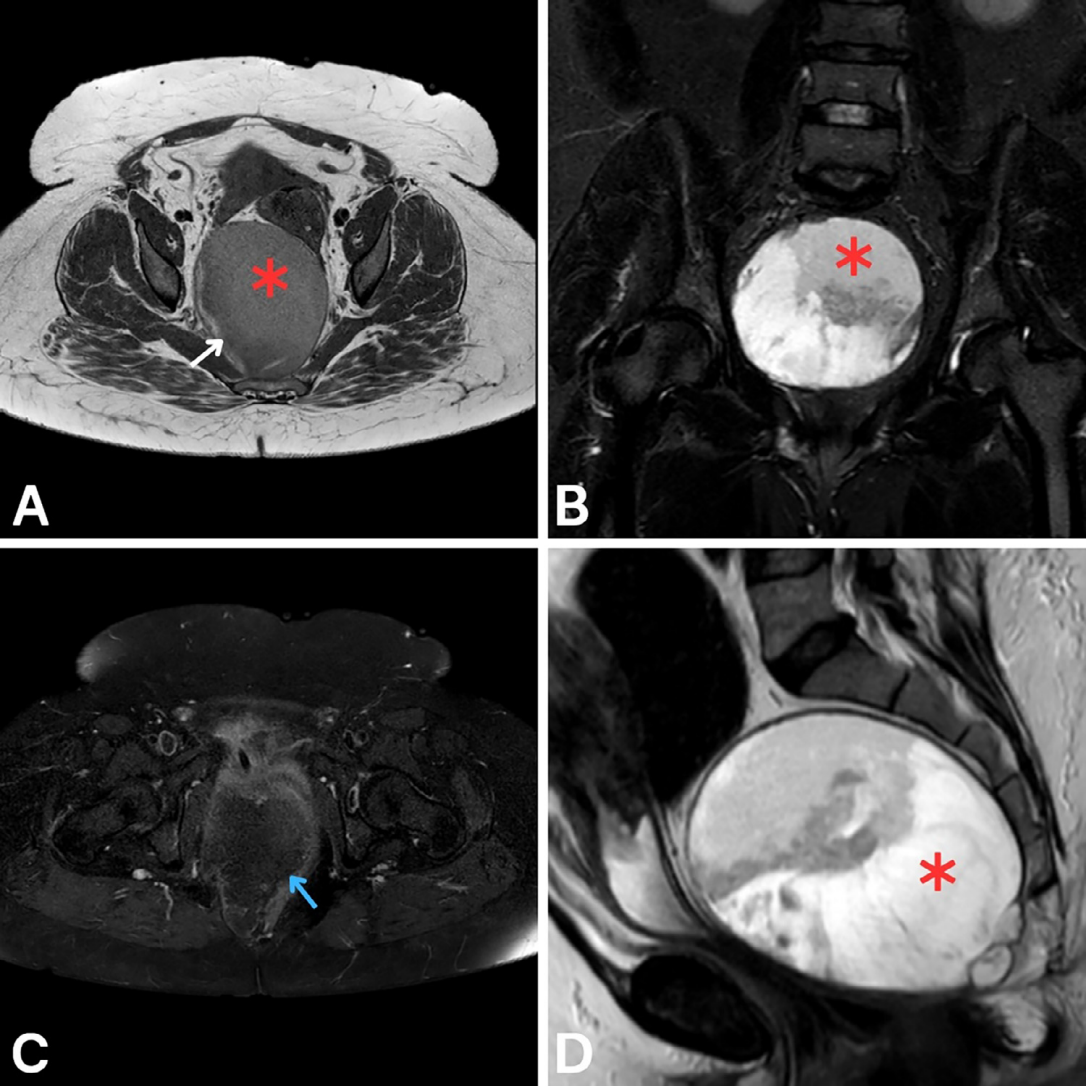
Initial pelvic MRI findings prior to and following intravenous gadolinium administration: (A) Axial T1-weighted image without contrast shows a hypointense presacral lesion with a well-defined wall (red asterisk) and areas of hyperintense signal suggestive of proteinaceous or hemorrhagic content (white arrow). (B) Coronal STIR image shows the lesion (red asterisk) with heterogeneous signal intensity, predominantly high. (C) Axial postcontrast T1-weighted fat-saturated image reveals peripheral nodular enhancement (blue arrow). (D) Sagittal T2-weighted image further illustrates the lesion (red asterisk) with heterogeneous high signal intensity.

Pelvic MRI revealed a well-defined, oval-shaped presacral lesion measuring 11 × 10 × 13 cm (anteroposterior × transverse × craniocaudal). Axial T1-weighted images showed a hypointense lesion with a well-defined wall and internal hyperintense areas suggestive of proteinaceous or hemorrhagic content (Fig. 1A). Coronal STIR and sagittal T2-weighted sequences showed heterogeneous signal intensity with predominantly high T2/STIR signal (Figs. 1B and D). Postcontrast T1-weighted fat-saturated imaging revealed peripheral nodular enhancement (Fig. 1C). The lesion exerted a significant mass effect on adjacent structures, including the rectum, uterus, and urinary bladder. No communication with the rectum or posterior neural involvement was observed.

The imaging findings, clinical symptoms, and elevated CEA levels raised suspicion for a presacral tailgut cyst. The absence of neural involvement on imaging ruled out neurogenic tumors, while the lack of communication with the rectum made an enteric duplication cyst less likely. Further imaging and histopathological analysis were necessary to confirm the diagnosis.

Given the lesion’s size and the mass effect on adjacent structures, the patient underwent exploratory laparotomy. During the procedure, there was an iatrogenic injury to the left iliac vein, which was repaired intraoperatively. Due to the patient’s unstable condition during surgery, only an incomplete resection of the cyst was performed. The lesion contained pus and gelatinous material, and subsequent histopathological examination confirmed that the cyst was benign. Three surgical drains were placed to manage postoperative drainage.

**Fig. 2:**
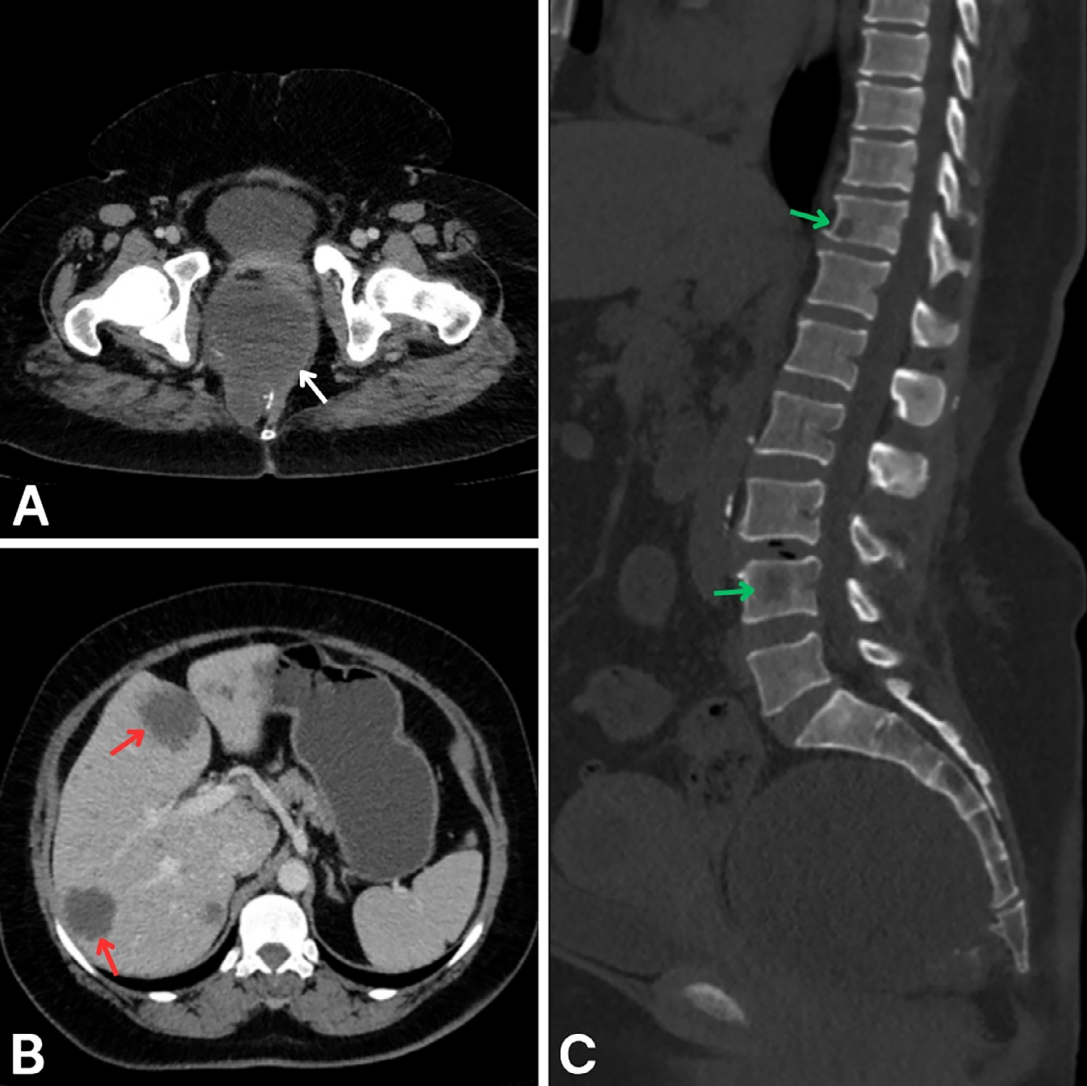
Follow-up abdominopelvic CT scan with and without intravenous contrast. (A) Axial CT at the pelvic level showing a presacral cystic lesion with peripheral nodular wall enhancement (white arrow). (B) Axial CT in the portal venous phase showing multiple hypodense hepatic lesions representing liver metastases (red arrows). (C) Sagittal CT in bone window showing lytic lesions at the D11 and L4 vertebral bodies, consistent with bone metastases (green arrows).

The patient’s postoperative course was initially uneventful, and she was advised to return for regular follow-up evaluations. However, she did not attend her scheduled follow-up visits and returned to our unit 6 months later with recurrent symptoms, including lower abdominal pain, urinary retention, and new-onset lower limb weakness. DRE identified a mass located approximately 3 cm from the anal verge. Abdominopelvic CT scan with and without intravenous contrast revealed a presacral midline cystic lesion measuring 10 × 10 × 12 cm with peripheral nodular wall enhancement, exerting a mass effect on the rectum and causing posterior scalloping of the sacrum (Fig. 2A). Additional imaging showed an enlarged liver with multiple hypodense lesions, the largest measuring 4 × 3.5 cm, consistent with hepatic metastases (Fig. 2B). Furthermore, sagittal bone window views revealed lytic lesions involving the D11 and L4 vertebral bodies, suggestive of osseous metastases (Fig. 2C).

**Fig. 3:**
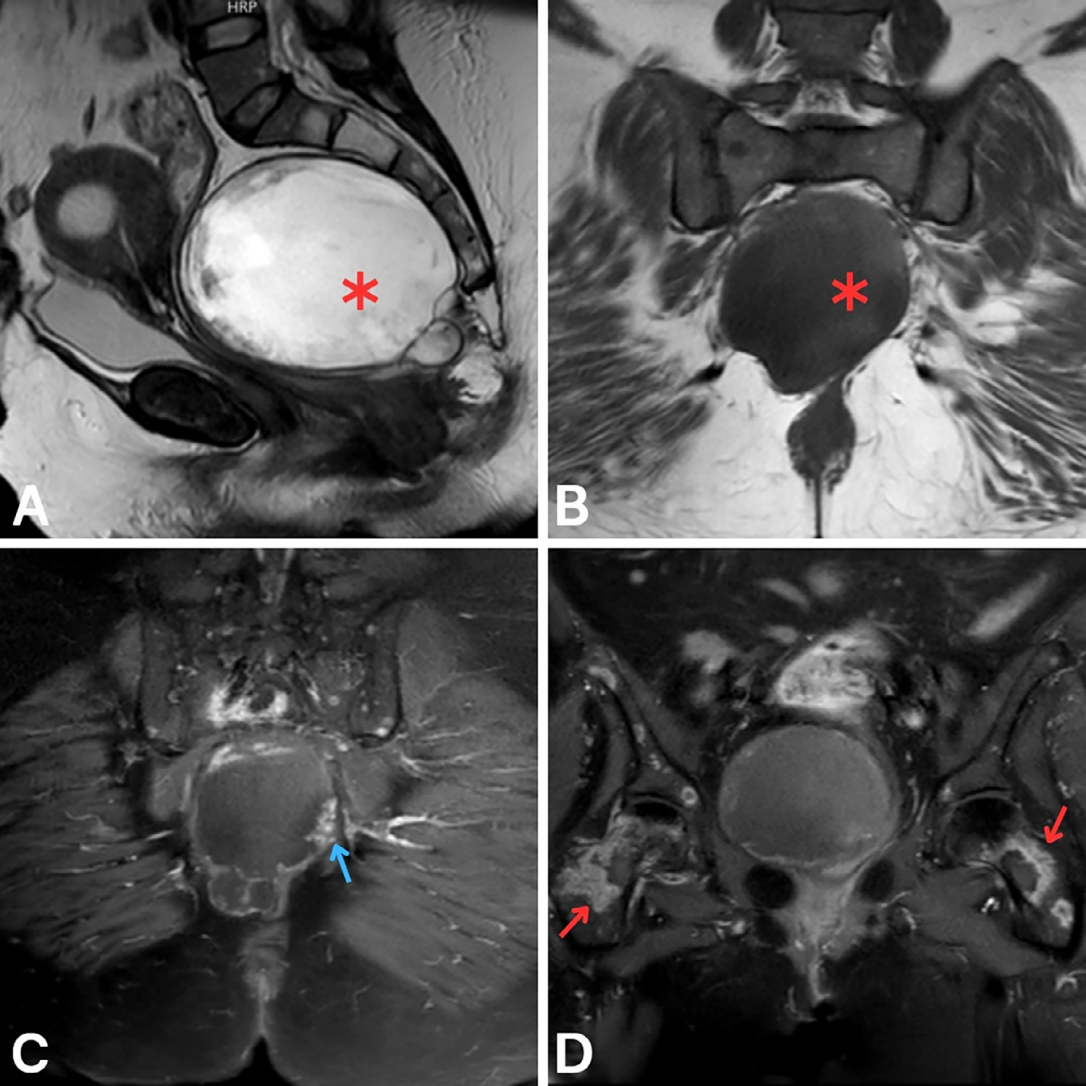
Follow-up pelvic MRI with and without intravenous gadolinium contrast. (A) Sagittal T2-weighted image showing a hyperintense presacral lesion with a well-defined wall (red asterisk). (B) Coronal T1-weighted image (precontrast) showing the presacral lesion as hypointense (red asterisk). (C,D) Coronal T1 fat-saturated images postcontrast showing peripheral nodular enhancement (blue arrow) and enhancing bony metastases (red arrows).

Further MRI imaging confirmed the presence of a well-defined cystic lesion in the presacral space, measuring 13 × 10.5 × 9.5 cm (Fig. 3A–D). On sagittal T2-weighted images, the lesion appeared hyperintense with a clearly defined wall. Coronal T1-weighted images without contrast showed low signal intensity. Postcontrast T1 fat-saturated sequences showed peripheral nodular enhancement, raising suspicion for malignancy. Additionally, enhancing bone lesions consistent with metastases were noted in the pelvis and vertebrae. A liver biopsy subsequently confirmed mucinous adenocarcinoma. Despite the initiation of chemotherapy, the disease progressed rapidly. The patient's condition progressively worsened, and she passed away 2 months after initiating chemotherapy.

## Discussion

This case describes a female patient who initially presented to our department and was found to have a large presacral TGC. Surgical excision was incomplete due to intraoperative complications, and histopathology confirmed a benign lesion. However, she returned 6 months later with recurrent symptoms and new-onset lower limb weakness. Imaging revealed a recurrent cystic mass with hepatic and bony metastases. A liver biopsy confirmed mucinous adenocarcinoma, indicating potential malignant transformation of the tailgut cyst.

TGCs are rare, but their potential for malignant transformation warrants careful monitoring. The clinical presentation of TGCs can vary greatly, depending on the size of the cyst and the organs it compresses [3]. In some cases, they may be asymptomatic and discovered incidentally on imaging, while in others, they can prolapse through the anus, potentially being misdiagnosed as hemorrhoids [3]. The compression of surrounding organs can lead to a wide range of symptoms, including urological, neurological, and defecation issues.

Malignant transformation of TGCs, although rare, remains a significant concern. A study on the surgical excision of TGCs revealed a higher-than-expected incidence of malignancy, with 13% of cases showing malignant degeneration, including adenocarcinomas and carcinoid tumors [6]. Notably, no specific clinical factors were found to predict malignancy, but imaging features like an enhancing solid component were observed in malignant cases [6]. Furthermore, 24 cases of malignancy arising from the epithelial lining of the cyst have been reported, emphasizing the necessity of careful histological examination of the surgical specimen [7]. In our case, the initially benign presentation of the tailgut cyst, followed by the development of liver metastases and mucinous adenocarcinoma, highlights the potential for malignant transformation over a short time.

In rare cases, TGCs can mimic other conditions like rectal cancer due to their location near the rectum and overlapping symptoms, including pain, bloating, and rectal pressure [3]. Our patient showed atypical features, such as new-onset tenesmus and altered bowel habits postsurgery, raising concerns about malignancy.

The diagnosis of TGCs relies heavily on imaging techniques such as MRI and CT scans. MRI typically shows TGCs as hypointense on T1-weighted images and hyperintense on T2-weighted images, though the appearance may vary depending on the cyst contents. CT scans often reveal presacral tumors with a central liquid component, peripheral tissue density, and fine calcification at the rim [8]. Histological examination remains the standard to confirm the diagnosis [4].

Surgical excision is the preferred treatment for both symptomatic and asymptomatic TGCs to prevent complications such as recurrence, infection, and malignant transformation [5]. The surgical approach is determined by the cyst's location: low-lying cysts are typically approached via a posterior route, such as the *Kraske* or parasacral incision, while higher cysts may require an anterior or transabdominal approach [3]. In certain cases, a combined abdominosacral approach is needed [9]. Ensuring complete removal with clear margins is crucial to minimize the risk of recurrence, and regular follow-up, including DRE and CT scans, is recommended. Recent advancements in laparoscopic techniques have enhanced the surgical options for low-lying cysts, providing better visualization in a narrow pelvic space for skilled surgeons [9]. Regarding coccygectomy, opinions vary. While some argue that coccygectomy helps reduce recurrence by improving exposure and eliminating potential cellular remnants [10]. Other studies indicate that it is unnecessary when the cyst is not attached to the coccyx [11].

Several reports have described malignant transformation in tailgut cysts. Cases have shown that elevated CEA levels and imaging features such as solid components or calcifications may raise suspicion for malignancy [12,13]. Surgical resection remains the primary treatment modality, with approaches varying based on tumor size and location. Some cases achieved clear margins with no recurrence following surgery and adjuvant therapy, while others experienced metastatic progression despite intervention [14,15].

## Conclusion

This case highlights the potential for malignant transformation of presacral TGCs. More research into the pathophysiology, diagnosis and management of these rare lesions is warranted.

## Ethical approval

Local ethics committees don’t require ethical approval to report such cases.

## Patient consent

The authors declare that they have obtained written informed consent prior to writing the article, including permission for publication of the images and clinical data included herein.
